# Advancing Repetitive Transcranial Magnetic Stimulation for Depression: Novel Protocols, Precision Targeting, and Future Directions

**DOI:** 10.31083/AP48750

**Published:** 2026-09-03

**Authors:** Wenzhong Liu, Yang Fan, Pingping Ma, Ting Song, Bo Liu, Kezhi Liu, Youguo Tan

**Affiliations:** ^1^Department of Psychiatry, The Affiliated Hospital, Southwest Medical University, 646000 Luzhou, Sichuan, China; ^2^School of Mental Health, Southwest Medical University, 643020 Zigong, Sichuan, China; ^3^Department of Psychiatry, Zigong Mental Health Center, 643020 Zigong, Sichuan, China; ^4^Center for Psychiatry Research, Zigong Institute of Brain Science, 643020 Zigong, Sichuan, China

**Keywords:** depression, major depressive disorder, treatment-resistant depression, neuromodulation, transcranial magnetic stimulation, precision medicine

## Abstract

Major depressive disorder (MDD) remains a significant global health challenge, with substantial limitations inherent in current pharmacotherapeutic and psychotherapeutic approaches. Repetitive transcranial magnetic stimulation (rTMS) has emerged as a safe, effective, and non-invasive neuromodulation therapy, particularly for treatment-resistant depression (TRD). However, conventional rTMS is limited by long treatment duration and variable interindividual efficacy. This expert opinion examines recent advances in rTMS optimization strategies aimed at overcoming these limitations. Key areas of progress include the development of novel stimulation protocols such as theta-burst stimulation (TBS); accelerated regimens and priming TMS; the use of magnetic resonance imaging (MRI)-guided TMS; and the integration of closed-loop systems using real-time electroencephalography (EEG) or functional near-infrared spectroscopy (fNIRS) feedback. Additionally, technological innovations, including robot-assisted TMS and wearable devices, promise to enhance targeting accuracy and treatment accessibility. Despite these developments, significant challenges impede clinical translation, including the high cost of personalized protocols, insufficient standardization and validation of novel approaches, and technical barriers to closed-loop implementation. Future efforts should focus on cost reduction, large-scale randomized controlled trials, biomarker development, and device portability. Ultimately, optimized rTMS shows significant potential to become a more efficient, precise, and accessible cornerstone of depression therapy.

## Main Points

1. Emerging transcranial magnetic stimulation (TMS) protocols, such as theta-burst stimulation (TBS) and accelerated regimens, not only shorten the overall treatment course but also enhance its efficacy.

2. Combined with magnetic resonance imaging (MRI)-guided targeting, rTMS achieves high remission rates in patients with treatment-resistant depression (TRD).

3. The development of real-time closed-loop repetitive transcranial magnetic stimulation (rTMS) technologies, such as rTMS-electroencephalography (EEG) and rTMS-functional near-infrared spectroscopy (fNIRS), represents a key advancement that will enhance targeting accuracy and individualized therapy.

4. Although promising, the efficacy and feasibility of these emerging precision rTMS strategies for depression require validation in large-scale clinical trials.

## 1. Introduction

Major depressive disorder (MDD) is a global public health issue characterized by high prevalence and high disability rates, exerting a devastating impact on patients’ quality of life and social functioning. The World Health Organization has ranked it as the third leading cause of the global disease burden and predicted that it will become the primary cause by 2030 [1,2]. Treating depression remains a significant challenge. Research has indicated that a substantial proportion of patients do not achieve adequate symptom relief after initial pharmacotherapy, and approximately 30% ultimately develop treatment-resistant depression (TRD), exhibiting inadequate response to multiple medication regimens [3]. Although psychotherapy has demonstrated some efficacy, its accessibility, cost, and requirements for both patients and therapists limit its widespread application [4]. Given the limitations of pharmacotherapy and the challenges of psychotherapy, developing safe and effective alternative or complementary therapies is essential.

Against this backdrop, physical therapies, particularly neuromodulation techniques, have achieved remarkable progress in depression treatment over recent decades. The spectrum of physical treatments has continuously expanded, ranging from the earliest electroconvulsive therapy (ECT), still considered the gold standard for TRD treatment but requiring anesthesia and having short-term cognitive side effects and significant stigmatization issues [5,6], to emerging techniques such as transcranial electrical stimulation (TES), magnetic seizure therapy (MST), vagus nerve stimulation (VNS), and deep brain stimulation (DBS). However, these therapies either require anesthesia and are invasive procedures (VNS, DBS), hindering widespread adoption [7,8], lack sufficient evidence for efficacy (TES), necessitating further validation [9], or face limitations in clinical application due to high costs (MST, DBS) [10,11]. However, in recent years, repetitive transcranial magnetic stimulation (rTMS) has gained increasing attention due to its unique advantages in the treatment of MDD.

rTMS is a neuromodulation technique that uses time-varying pulsed magnetic fields to induce electrical currents within specific brain target regions (primarily the dorsolateral prefrontal cortex, DLPFC), thereby modulating neuronal excitability and brain-network function [12,13,14]. Recent evidence has further suggested that these neuroplastic effects could be mediated by the neurotrophin system, specifically through the upregulation of brain-derived neurotrophic factor (BDNF), which plays a crucial role in stabilizing mood and enhancing therapeutic outcomes [15]. Owing to its non-invasiveness, favorable safety profile, and increasingly well-established efficacy, rTMS has emerged as a significant treatment option for depression since its approval by the United States Food and Drug Administration (FDA) for treating adult depression in 2008 [16,17]. Substantial evidence, including high-quality meta-analyses, has demonstrated that rTMS treatment is significantly superior to sham stimulation in alleviating depressive symptoms, even demonstrating efficacy in a subset of pharmacotherapy-resistant patients [18,19]. The standard rTMS treatment protocol (e.g., high-frequency stimulation of the left DLPFC) has been incorporated into several international clinical guidelines. It has been recommended as a first- or second-line treatment for mild-to-moderate depression and for the treatment of TRD [16,20,21]. Common side effects (e.g., transient headache, scalp discomfort) are generally mild, and the incidence of serious adverse events (e.g., seizures) is <0.6% [22]. Notably, the therapeutic potential of rTMS has been validated in multiple diseases with depressive manifestations. In patients with post-traumatic stress disorder (PTSD), high-frequency rTMS not only improved PTSD core symptoms but also significantly alleviated the frequently accompanying depression, with advantages of high compliance and low adverse reactions [23,24]. In patients with post-stroke depression (PSD), rTMS improved depression scores, cognitive function, and activities of daily living, further confirming the reliability of non-invasive brain stimulation (NIBS) in depression-related conditions [25]. These findings provided a solid clinical basis for the optimized application of rTMS in MDD and also highlighted its broad applicability potential as a non-pharmacological therapy.

Historically, seminal meta-analyses indicated conservative response rates of approximately 29% for standard protocols [26]. More recent meta-analyses of randomized controlled trials have reported improved outcomes, with response rates reaching approximately 40% and remission rates around 35% [27]. Real-world observational studies reported even higher effectiveness, with response rates ranging from 58% to 83% in clinical-practice settings [28]. However, despite these advances, a comparative efficacy gap remained when those results were compared to the emerging accelerated protocols discussed later in this review. In recent years, the research focus in the rTMS field has shifted from solely validating efficacy towards the in-depth optimization of treatment parameters and protocols. This shift aimed to overcome the limitations of the standard protocol and to enhance treatment efficiency, efficacy, and individualization [29,30,31]. Significant advances have emerged, including novel stimulation protocols, personalized treatment approaches, closed-loop stimulation systems, and device innovations [30,32]. Despite the flourishing research in rTMS optimization and its considerable potential, the efficient and cost-effective translation of these laboratory findings into widely accessible clinical practice faces prominent challenges [33].

The primary objective of this review was to focus on rTMS as an important non-invasive physical treatment modality for depression. We examined the latest progress in its optimization research, encompassing innovations in stimulation protocols, precision treatment strategies, closed-loop systems, and device development. Furthermore, we critically analyzed the key challenges currently impeding clinical translation. This review explored viable pathways for bridging the gap between laboratory research and clinical practice and provided insights into overcoming these obstacles. Ultimately, we offered perspectives that may enhance the clinical efficacy, efficiency, and accessibility of rTMS, thereby facilitating its evolution from a significant complementary therapy into an efficient, precise, and accessible cornerstone of depression therapy.

## 2. rTMS: Optimization and Clinical Translation

rTMS is a widely utilized non-invasive physical therapy for depression. Its efficacy and safety profile have been substantiated through years of clinical application [14,22]. In recent years, significant research advances within the rTMS field have shown its considerable development potential and promising future prospects. Should these research findings be effectively translated into clinical practice, rTMS holds the potential to become a major therapy for depression treatment. Optimization of rTMS primarily involves parameter refinement, novel stimulation protocols, refinement of therapeutic targets, and algorithm-assisted treatment strategies. The following sections examined relevant research findings and their translation into clinical applications across those domains.

### 2.1 Standard Protocol

The most commonly used standard rTMS protocol for depression treatment, as recommended by guidelines (e.g., Canadian Network for Mood and Anxiety Treatments [CANMAT] and the American Psychiatric Association [APA]), involves targeting the left DLPFC using a figure-of-eight (Fo8) coil. Stimulation parameters typically include an intensity of 110–120% of the resting motor threshold (RMT), with 120% RMT currently considered the standard target dose to maximize clinical efficacy, a frequency of 10 Hz, with each train lasting 4 s, an inter-train interval of 26 s, repeated 75 trains/session, resulting in a total of 3000 pulses/day. Treatment is administered 5 days/week for a duration of 4 to 6 weeks [20,21].

A low-frequency (1 Hz) stimulation protocol targeting the right DLPFC is also used. The fundamental difference between these approaches lies in their neuromodulatory effects: high-frequency stimulation (>5 Hz) is generally considered to increase cortical excitability, whereas low-frequency stimulation (1 Hz) tends to decrease cortical excitability [34]. A meta-analysis by Chen et al. [35] indicated that right-sided low-frequency stimulation demonstrated efficacy comparable to the standard protocol and apparently induced fewer side effects. Furthermore, bilateral rTMS protocols, combining high-frequency stimulation over the left DLPFC with low-frequency stimulation over the right DLPFC, are sometimes used clinically. Although earlier meta-analyses suggested that bilateral rTMS might not confer significant efficacy advantages over unilateral stimulation [36], more recent evidence has indicated that it may offer benefits for specific clinical profiles, particularly in patients with depression and comorbid anxiety [37]. Consequently, although standard left DLPFC stimulation remains the first-line choice, bilateral protocols serve as a valuable alternative in complex cases.

### 2.2 Coil Selection

Transcranial magnetic stimulation uses coils of various shapes. The Fo8 coil and the H-coil are currently the most commonly used FDA-approved types. The Fo8 coil generates a more focal stimulus, whereas the H-coil provides greater stimulation depth and a broader field of coverage—a technology widely known as deep TMS (dTMS) [38,39,40]. Research has shown that with the same stimulation frequency, the H1 coil (a type of H-coil designed for high-intensity, broad targeting) may be more effective in alleviating depressive symptoms than is the more focally oriented Fo8 coil, but clinical trials directly comparing the efficacy of different coil shapes are still lacking [41,42].

It is important to note that there is an inverse relationship between the focality of a coil and its depth of penetration. Among current commercially available coils, the double-cone coil offers relatively high energy efficiency and achieves a favorable balance between stimulation depth and superficial field strength [43,44]. The optimal approach for coil selection may involve choosing the most suitable coil based on specific treatment requirements or developing novel coils capable of adaptable shaping.

### 2.3 Stimulation Intensity

The most common method for determining rTMS stimulation intensity is the assessment of the patient’s motor threshold (MT). MT is defined as the minimal intensity of motor cortex stimulation required to elicit a reliable motor evoked potential (MEP) of minimal amplitude (typically >50 μV) in the target muscle (e.g., abductor pollicis brevis) in at least 50% of trials [45]. It can also be assessed visually as the minimal intensity required to produce a visible twitch in the contralateral hand muscle [46]. Intensity is usually expressed as a percentage of the maximum stimulator output (MSO) [47]. The RMT is determined while the target test muscle is at rest, and active motor threshold (AMT) is usually determined during a slight tonic contraction of the target muscle at approximately 20% of the maximal muscle strength [45]. AMT was, on average, at 82% of RMT [48]. For the standard rTMS protocol, intensities typically range from 110% to 120% RMT [49]. In addition, standard TBS typically uses a lower intensity, set at 80% of the AMT rather than the RMT, due to its unique burst-firing pattern and higher pulse frequency.

However, studies have indicated that the MT can vary over the course of treatment [50]. Furthermore, individual differences in skull morphology, degree of brain atrophy, and other anatomical factors can lead to variations in the depth and cortical excitability of the target region [51,52]. These variations limit the precision of this traditional MT-based method for determining the optimal patient-specific stimulation intensity [53,54]. Additionally, disease severity and patient tolerance also influence the selection of stimulation intensity.

To address these limitations, one potential solution involves integrating individual magnetic resonance imaging (MRI) data with computational modeling (e.g., SimNIBS, ROAST) to simulate the distribution of the electric field (E-field) within the brain, thereby identifying the optimal stimulation site and intensity [55]. Compared to MT-based approaches, E-field modeling for individualized parameter optimization can reduce errors in stimulation dosage arising from anatomical variations [56]. Another prominent optimization strategy uses rTMS combined with electroencephalography (rTMS-EEG). This technique enables the measurement and assessment of cortical network excitability and connectivity [57]. By providing real-time readouts such as TMS-evoked potentials (TEPs) from the target brain region, rTMS-EEG facilitates operator adjustment of stimulation intensity [58,59,60]. Consequently, rTMS-EEG technology is also being used to develop individualized EEG biomarkers for assessing treatment response and aiding in the implementation of closed-loop brain-stimulation systems.

### 2.4 Target Selection and Localization Methods

The conventional target for rTMS treatment of depression is the left DLPFC. The right or bilateral DLPFC is also commonly used as a stimulation target in clinical practice. With the deepening understanding of the neurobiological mechanisms underlying depression, researchers have proposed several novel potential targets for TMS therapy, such as the dorsomedial prefrontal cortex (DMPFC) [61], frontopolar cortex (FPC) [62], the ventromedial prefrontal cortex (VMPFC), and the ventrolateral prefrontal cortex (VLPFC) [63], as well as the orbitofrontal cortex (OFC) [64] and the visual cortex (VC) [65]. Several rTMS studies targeting the DMPFC have demonstrated promising results in terms of safety and efficacy [61,66,67]. However, a double-blind, placebo-controlled trial showed no significant difference in efficacy between DMPFC-rTMS and sham stimulation [68]. Regarding the OFC (which plays a key role in reward processing and non-reward punishment systems), one study found that 1 Hz right-sided OFC-rTMS appeared to be safe and tolerable, and to alleviate symptoms in MDD patients where conventional rTMS has failed [69]. Furthermore, clinical trials targeting the VLPFC and VC have also shown therapeutic potential through functional magnetic resonance imaging (fMRI) measurements [70,71]. In summary, although several novel targets are supported by neurobiological rationale, and preliminary studies showed some clinical promise, they all lack validation and broader application through large-scale clinical trials [63].

Regarding target localization, there are various targeting methods for rTMS in depression (Table 1). A standard approach is to use the international 10-20 EEG system, determining the location through measurement and calculation based on the subject’s head anatomy. This method is often simplified by using a standard 10-20 EEG cap to identify the target area (e.g., the DLPFC corresponds to the F3 electrode position). This method is low-cost and offers a reasonable degree of accuracy [72]. However, it is important to note that the localization accuracy of the 10-20 system is inferior to that of neuroimaging-based neuronavigation techniques [72,73]. Additionally, applying the 10-20 system requires operators to possess certain anatomical knowledge and measurement skills. This has posed practical difficulties and has led to the proposal of simplified methods for locating the F3 position, known as the Beam F3 method [74]. That method locates the F3 EEG site using a simpler set of measurements that can be quickly implemented in a clinical setting, with high reliability [75,76]. Moreover, the earliest and most convenient method for locating the DLPFC involves identifying the primary motor cortex (M1) area for the hand, typically by eliciting MEPs in a target muscle like the abductor pollicis brevis, and then moving 5 cm anteriorly [77,78]. That method is simple, rapid, and inexpensive, but it does not account for individual anatomical variations, resulting in suboptimal precision [79,80].

**Table 1.** **Comparison of targeting methods for rTMS in depression**.

Method	Validity	Cost	Operational difficulty
International 10-20 EEG system	Moderate (Reasonable accuracy; fails to account for individual anatomical/functional variability)	Low-cost	Moderate; requires anatomical knowledge and measurement skills
Beam F3 method	Moderate (High reliability; simplified for clinical application; inherits limitations of 10-20 system)	Low-cost	Low; simplified measurement protocol, rapid implementation in clinical settings
M1-based anterior 5 cm method	Low (Suboptimal precision; does not consider individual anatomical variations)	Low-cost	Low; simple and rapid operation
fMRI-guided localization	High (High anatomical/functional specificity; targets individual optimal site; associated with better clinical outcomes)	High-cost	High; time-consuming; requires expertise in neuroimaging analysis and specialized algorithms
DTI-guided navigation/sMRI-guided navigation	Clinical efficacy and precision remain unvalidated	High-cost	High; requires expensive equipment and complex operational procedures

Notes: (1) Validity assessment is based on anatomical accuracy, individualization adaptability, and reported clinical relevance in the literature. (2) Cost classification: Low-cost = No specialized imaging equipment required; High = Dependent on advanced neuroimaging (fMRI/DTI/sMRI) or specialized devices. (3) Operational Difficulty classification: Low = Simple measurements/protocols (rapid implementation); Moderate = Requires basic professional skills; High = Requires specialized expertise (neuroimaging analysis) or complex workflows. Abbreviation: rTMS, repetitive transcranial magnetic stimulation; EEG, electroencephalography; M1, primary motor cortex; F3, the left frontal 3 electrode site in the international 10-20 system; fMRI, functional magnetic resonance imaging; sMRI, structural magnetic resonance imaging; DTI, diffusion tensor imaging.

Crucially, even disregarding the anatomical inaccuracies inherent in these two mainstream localization methods, individual variability in functional neurocircuitry can also lead to inconsistent clinical responses [81,82]. Therefore, seeking a single, standardized anatomical location cannot fully resolve the challenge of target localization. To address these issues, a more effective approach involves using neuroimaging techniques for localization based on functional connectivity to find the optimal treatment target for the individual patient [83]. The strategy involves acquiring fMRI scans of the patient’s brain and using algorithms to identify the location within the individual’s left DLPFC that demonstrated the strongest negative functional connectivity with the subgenual anterior cingulate cortex (sgACC), which serves as the treatment target and is associated with better clinical outcomes [81,84,85]. Furthermore, recent studies have explored diffusion tensor imaging (DTI)-guided and structural magnetic resonance imaging (sMRI)-guided navigation approaches for non-invasive neuromodulation [86,87,88]. But these methods still require expensive equipment and complex operational procedures, and their clinical efficacy remains to be further investigated and validated.

Considering the significant time and financial costs, the widespread clinical implementation of highly individualized precision targeting remains challenging. Although studies by Cash et al. [83] and the success of the Stanford Accelerated Intelligent Neuromodulation Therapy (SAINT) protocol highlighted the critical importance of functional connectivity-based targeting for maximizing efficacy, future research must also focus on developing cost-effective technologies (such as novel E-field modeling [89,90]) to make this precision accessible. Alternatively, in resource-limited settings, optimizing other parameters (e.g., dose, frequency) remains a pragmatic strategy.

## 3. Novel Treatment Protocols

As previously mentioned, the standard rTMS protocol (Fig. 1A) has notable limitations: a meta-analysis indicated that its response rates are 39.7% and remission rates around 35% [27]. Given that the standard regimen requires daily sessions of 37.5 min over 4–6 weeks, those outcomes are suboptimal. By modifying the adjustable parameters of TMS, several novel protocols have been developed since the introduction of standard rTMS to optimize and improve upon conventional treatment shortcomings (Table 2, Ref. [16,20,21,26,27,35,36,91,92,93,94,95,96,97,98,99,100,101,102,103,104,105,106,107,108]).

**Fig. 1.**
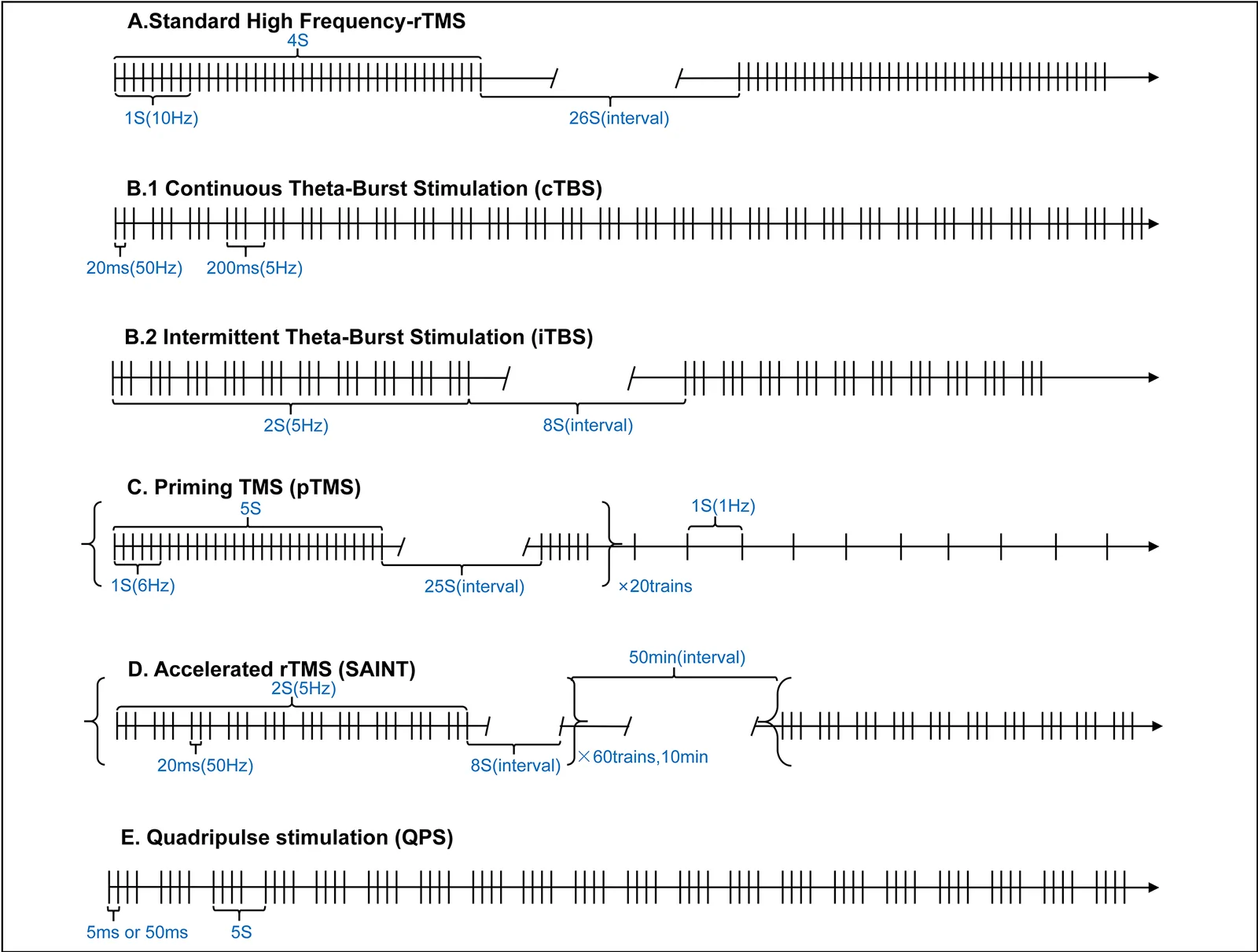
**Schematic diagrams of different rTMS protocols for MDD**. (A–E) illustrate the key parameters of a specific rTMS protocol, including stimulation frequency, intensity, inter-train interval (ITI), total number of pulses/session, and total treatment duration/session. (A) Standard High-Frequency rTMS: Targets the left DLPFC with a stimulation frequency of 10 Hz and intensity of 110–120% RMT. Each session consists of 75 trains (40 pulses per train) with a 26-s ITI, resulting in 3000 total pulses and a total session duration of 37.5 min. (B.1) Continuous Theta-Burst Stimulation (cTBS): Based on the original experimental protocol developed for motor-cortex physiology (e.g., Huang et al., 2005 [110]), the intensity is set at 80% of the active motor threshold (AMT). Stimulation is delivered as continuous bursts (3 pulses/burst at 50 Hz, repeated every 200 ms [5 Hz burst frequency]), totaling 600 pulses/session with a 40-s total duration. (B.2) Intermittent Theta-Burst Stimulation (iTBS): The experimental paradigm also uses 80% AMT; however, the FDA-cleared clinical protocol for major depressive disorder (e.g., based on the THREE-D trial) typically employs an intensity of 120% of the resting motor threshold (RMT). It features the same 50 Hz intraburst frequency but with intermittent delivery (2-s stimulation trains followed by 8-s ITIs). Each session includes 20 trains (10 bursts/train, 3 pulses/burst), resulting in 600 total pulses and a 190-s total duration. (C) Priming TMS (pTMS): Combines a preconditioning (priming) phase and a main stimulation phase. The priming phase uses 6 Hz stimulation (30 pulses/train, 20 trains, 25-s ITI) for 10 min (600 total priming pulses), followed by 1 Hz low-frequency rTMS (1800 s of stimulation). (D) Accelerated rTMS (SAINT): An intensive, fMRI-guided protocol targeting the left DLPFC (selected based on the strongest negative functional connectivity to the sgACC). Each session delivers 1800 pulses (3 pulses/burst at 50 Hz, 10 bursts/train, 60 trains with 8-s ITIs) over 10 min. Ten sessions are administered daily with 50-min inter-session intervals, totaling 90,000 pulses over 5 consecutive days. (E) Quadripulse stimulation (QPS): Uses 90% AMT and 4 monophasic pulses/train (5 ms or 50 ms inter-pulse intervals). Each 30-min session includes 12 trains/min (5-s ITI), resulting in 1440 total pulses. Abbreviations: cTBS, continuous theta-burst stimulation; iTBS, intermittent theta-burst stimulation; SAINT, Stanford Accelerated Intelligent Neuromodulation Therapy; MDD, major depressive disorder; sgACC, subgenual anterior cingulate cortex.

**Table 2.** **Comparison of different rTMS protocols for major depressive disorder**.

Protocol type	Stimulation parameters	Efficacy data	Advantages	Disadvantages	Evidence level*
Standard high-frequency (left DLPFC)	10 Hz,110%–120% of RMT, 3000 pulses/session, 4–6 weeks, 5 sessions/week	Response: ~40%; Remission: ~35% [27]	Well-established, guideline-recommended, non-invasive	Long treatment duration, moderate efficacy, interindividual variability	Strong (Multiple RCTs and meta-analyses [16,20,21,26])
Low-frequency (right DLPFC)	1 Hz, 110–120% RMT, similar pulse count and duration	Comparable to standard HF (30%–40%) [35]	Fewer side effects, well-tolerated	Limited clinical data, less standardized	Moderate (Fewer RCTs, meta-analysis support [35])
Bilateral rTMS	HF left DLPFC + LF right DLPFC	Variable; inconsistent superiority over unilateral (30%–40%) [36]	Theoretical dual modulation	Increased session time, alternative in complex cases	Moderate (Less evidence, lower guideline recommendation [16,36])
Theta burst stimulation (TBS)	50 Hz bursts, 5 Hz train, 80% AMT (experimental) or 120% RMT (clinical MDD protocol), 600 pulses/session	Non-inferior to standard HF rTMS (Response: ~40%–50%) [91]	Short session time, non-inferior efficacy	Requires AMT measurement, less long-term data	High (RCTs and meta-analyses [91,92,93,94])
Accelerated rTMS/TBS	Multiple sessions/day, total pulses not fixed	Comparable to standard protocols [95,96], some highly effective (Remission: ~79%–91% in specific trials) [97,98]	Shorter overall treatment duration	Protocol variability, limited RCTs, more resource-intensive per day	Moderate (Growing evidence, but heterogeneous protocols [95,96,99,100])
Priming TMS (pTMS)	Pre-conditioning (e.g., 6 Hz) + main stimulation (e.g., 1 Hz)	Promising in theory and network meta-analysis [101,102,103,104]	Potentiates treatment effect, may enhance efficacy	Limited RCTs, protocol not standardized	Low (Few RCTs, preliminary evidence [104,105])
Quadripulse stimulation (QPS)	90% AMT, 4-pulse trains, ISIs of 5 ms (QPS5) or 50 ms (QPS50), 1440 pulses/session	Preclinical/limited clinical data [106,107,108]	Potentially stronger and more stable plasticity induction	Requires specialized equipment, very limited clinical evidence	Low (few clinical trials [107,108])

Abbreviation: DLPFC, dorsolateral prefrontal cortex; TBS, theta-burst stimulation; pTMS, priming TMS; QPS, quadripulse stimulation; RMT, resting motor threshold; AMT, active motor threshold; ISI, inter-stimulus interval; HF, high-frequency; LF, low-frequency; RCT, randomized controlled trial; FDA, United States Food and Drug Administration. * Notes on Evidence Level Grading Criteria: The evidence levels presented in this table were systematically categorized based on the following operational framework tailored for this expert opinion:Strong: Supported by extensive evidence, including multiple high-quality randomized controlled trials (RCTs), robust comprehensive meta-analyses, regulatory approvals (e.g., FDA), and broad endorsement as a first-line or standard therapy by major international clinical guidelines (e.g., CANMAT, APA). High: Supported by multiple RCTs and meta-analyses demonstrating clear efficacy (e.g., non-inferiority to standard protocols) and possessing regulatory clearance (e.g., FDA), but potentially with less extensive long-term real-world data or historical precedence compared to “Strong” evidence. Moderate: Supported by growing clinical evidence from fewer or smaller RCTs, emerging meta-analyses, or large open-label trials. These protocols demonstrate clinical potential and theoretical validity but currently lack universal standardization or broad first-line recommendations in major guidelines. Low: Supported primarily by preliminary evidence, such as theoretical rationale, preclinical studies, network meta-analyses without sufficient direct head-to-head trials, or a very limited number of small-scale clinical trials. Requires significant large-scale validation before widespread clinical translation.

### 3.1 Theta-Burst Stimulation (TBS)

Theta-burst stimulation (TBS) is a brief TMS protocol approved by the FDA for TRD [109]. The original experimental protocol involves stimulation at 80% of the AMT, delivered in bursts of three pulses at 50 Hz, repeated every 200 ms (5 Hz), totaling 600 pulses/session [110]. In contrast, the FDA-cleared clinical iTBS protocol for TRD typically utilizes an intensity of 120% of the resting motor threshold (RMT) [91]. Two main paradigms are used: continuous TBS (cTBS), which involves uninterrupted stimulation (600 pulses in 40 s) and is thought to suppress cortical excitability (Fig. 1B.1, Ref. [110]), and intermittent TBS (iTBS), which consists of 2 s trains interleaved with 8 s intervals (600 pulses in 190 s) and is considered to enhance cortical excitability [109,110] (Fig. 1B.2). A 2018 multicenter non-inferiority trial (*n* = 414) demonstrated that iTBS, with a total daily treatment time of ~3 min, was not inferior to traditional high-frequency rTMS (37.5 min/session) for TRD [91]. Moreover, multiple meta-analyses have shown that TBS had comparable efficacy and tolerability to conventional rTMS, with a significant advantage in time efficiency [92,93,94]. 

### 3.2 Priming TMS (pTMS)

Priming TMS (pTMS) involves delivering a preconditioning stimulus before the main rTMS session in order to enhance treatment effects. This approach uses the modulatory effects of priming on cortical excitability [101,105,111]. A common pTMS protocol applies a 6-Hz priming stimulation to the right DLPFC followed by a 1-Hz therapeutic rTMS, which has been shown to induce long-term depression (LTD), reduce cortical excitability, and potentiate treatment outcomes [101,102] (Fig. 1C). Given its inhibitory effects, cTBS has also been investigated as a priming technique; studies have confirmed that cTBS priming reduced MEPs and may be used in the development of novel protocols [103,112]. A 2019 network meta-analysis comparing 18 non-surgical brain-stimulation techniques reported that only two ECT protocols outperformed pTMS in both response rate and acceptability, whereas bilateral rTMS, iTBS, and standard rTMS were less effective [104]. However, given the limited number of direct head-to-head trials and the wide confidence intervals in network meta-analyses, those results regarding pTMS should be interpreted with caution pending further large-scale validation.

### 3.3 Accelerated rTMS/TBS

Although standard rTMS has demonstrated efficacy in MDD, its time-intensive nature limits clinical applicability. Accelerated protocols are used to shorten the treatment duration while maintaining or enhancing efficacy by administering multiple daily sessions (2–5 sessions) over a shorter period (typically 5–10 days, though some protocols extend to two weeks), with the total number of pulses comparable to standard rTMS [99,100]. Recent meta-analyses and systematic reviews suggested that accelerated rTMS/TBS was comparable to standard protocols in efficacy, safety, and tolerability [95,96]. However, due to variations in accelerated-protocol parameters and a limited number of RCTs, those findings require confirmation through larger, well-designed studies [99,113]. A notable example of an accelerated therapy is the Stanford Accelerated Intelligent Neuromodulation Therapy (SAINT), an iTBS protocol that uses intensive parameters: 10 sessions/day for 5 consecutive days (1800 pulses/session, 50-min intervals), targeting the left DLPFC site with the strongest negative functional connectivity to the sgACC, incorporated fMRI-guided targeting and high-dose stimulation (90,000 pulses over 5 days) [114] (Fig. 1D). In an open-label trial, it achieved a 90.5% remission rate [97], and a subsequent double-blind trial reported a 78.6% remission rate [98]. These pivotal trials, characterized by large effect sizes, have led to FDA clearance for the SAINT protocol, pointing out the transformative potential of optimized, high-dose rTMS to significantly enhance treatment efficacy and speed [115].

### 3.4 Quadripulse Stimulation (QPS)

Quadripulse stimulation (QPS) is a novel stimulation protocol in which the stimulation intensity is typically set at 90% of the AMT. Each stimulus train consists of four monophasic TMS pulses, with inter-pulse intervals of either 5 ms or 50 ms. Trains are delivered every 5 s over a 30-min session, resulting in a total of 1440 pulses/session (4 pulses/train × 12 trains/min × 30 min) [116] (Fig. 1E). QPS with a short inter-stimulus interval (QPS5) induces long-term potentiation (LTP)-like effects and increases cortical excitability, whereas QPS with a long interval (QPS50) induces LTD-like effects and decreases cortical excitability [106,107]. Comparative studies have suggested that QPS may elicit more robust and stable synaptic plasticity than does TBS, suggesting its potential for therapeutic development [108]. However, the implementation of QPS requires specialized custom equipment [107], which limits its scalability and widespread adoption. Furthermore, clinical evidence supporting its efficacy remains scarce.

### 3.5 Other Emerging Protocols

Beyond the relatively well-established protocols, the flexibility of TMS parameters has spurred the development of various other innovative approaches, including synchronized TMS (sTMS). sTMS uses the application of low-intensity magnetic stimulation synchronized with individual alpha frequency [117]. This suggests that it may be effective, safe, and well tolerated [118,119,120]. Another emerging protocol is paired-pulse TMS (ppTMS), which delivers pairs of pulses with specific inter-stimulus intervals [121]. However, a 2017 network meta-analysis indicated that novel rTMS interventions (including accelerated, synchronized, and deep rTMS) were not significantly more effective than sham stimulation, and current evidence was insufficient to support the use of either sTMS or rTMS as standard treatments for MDD [122]. Many novel protocols still lack sufficient clinical-trial evidence, due not only to their recent development but also to scalability issues. For example, SAINT requires fMRI-guided individualized targeting, and QPS and ppTMS depend on specialized devices. These factors limit large-scale validation and clinical implementation. Therefore, ideal novel therapies should consider not only efficacy and safety but also accessibility by trying to minimize overall operational costs.

## 4. Future Directions

### 4.1 Closed-Loop rTMS

Conventional rTMS typically uses open-loop, fixed protocols, applying the same parameters across all individuals regardless of interindividual differences or dynamic brain states. Although practical, this approach may compromise treatment efficacy. Many studies use personalized rTMS parameters based on anatomical and functional neuroimaging, as discussed earlier, but the brain state at the time of stimulation also plays a crucial role in modulating the response to rTMS [123,124,125]. Therefore, closed-loop rTMS, which dynamically adapts stimulation parameters based on real-time neural activity, holds promise for enhancing treatment outcomes [123,126]. The primary challenge in implementing closed-loop stimulation is identifying reliable biomarkers that accurately reflect brain state and are reproducible across individuals, as demonstrated in previous biomarker research for DBS in depression [127]. Subsequently, rTMS parameters must be adjusted in real time based on these biomarkers. The development of rTMS-EEG technology has laid the foundation for such systems, the biomarkers for closed-loop rTMS are typically based primarily on rTMS-EEG-related research [123]. Recently, several studies have successfully implemented experimental closed-loop rTMS based on real-time EEG feedback by using sophisticated real-time signal analysis algorithms [128,129,130] (Fig. 2). Beyond EEG, the integration of functional near-infrared spectroscopy (fNIRS) with TMS is also emerging. Compared to fMRI, fNIRS is more portable and can be used to assess TMS treatment effects [131,132,133]. It is important that optical signals are unaffected by electromagnetic fields, thereby enabling simultaneous rTMS stimulation and fNIRS monitoring [134,135]. Although evidence is still limited, rTMS-fNIRS integration offers a promising alternative pathway toward closed-loop neuromodulation [133,136]. Overall, closed-loop systems represent a future direction for rTMS with significant potential. However, challenges such as biomarker validation and algorithmic complexity currently hinder clinical translation. Personalized closed-loop therapeutic TMS remains a long way off before it becomes a viable clinical option.

**Fig. 2.**
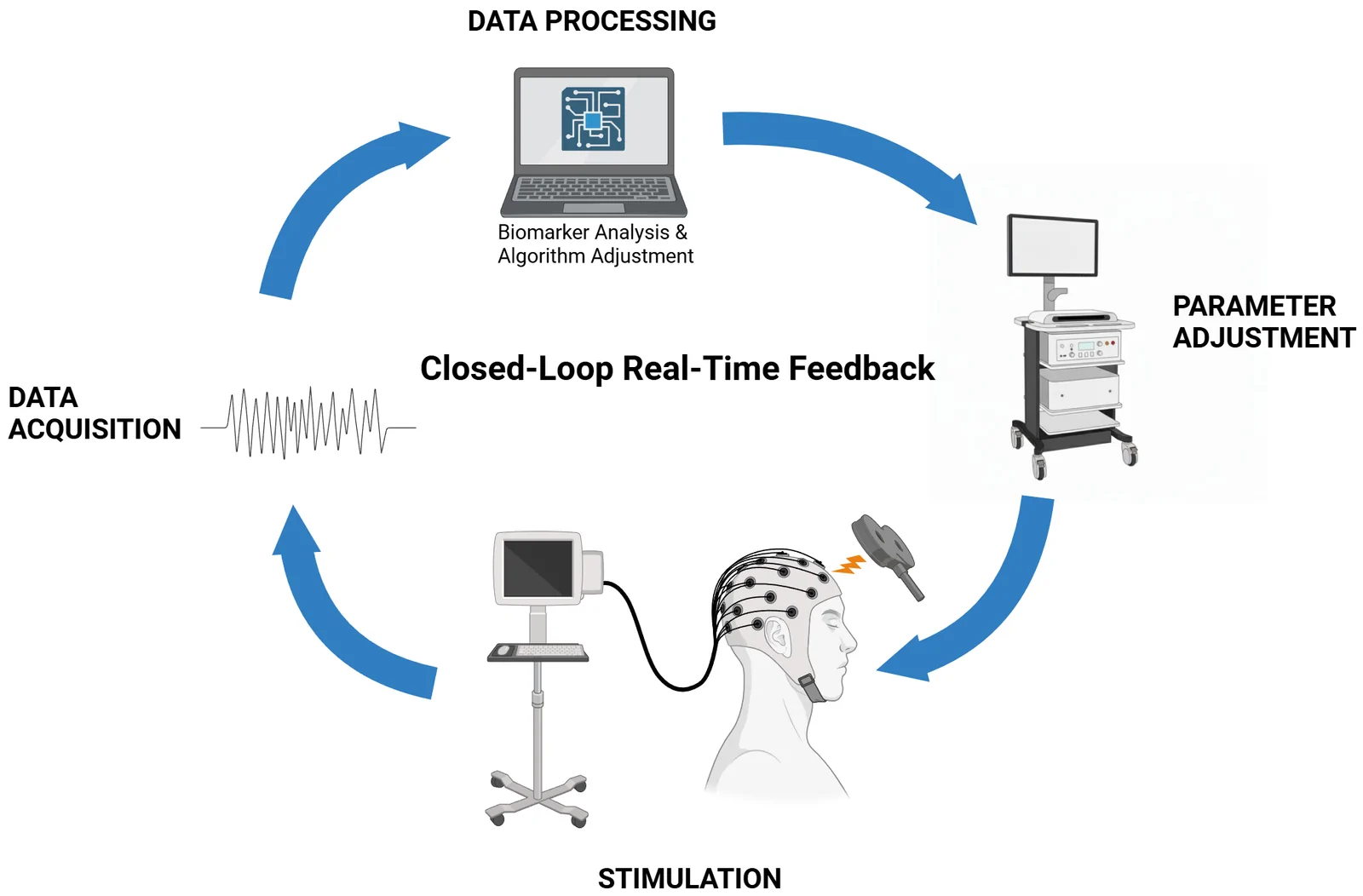
**Schematic diagrams of real-time closed-loop rTMS**. The TMS pulses are delivered via a magnetic coil positioned over the target area, while simultaneously, EEG and fNIRS monitor cortical activity, capturing TEPs and changes in hemoglobin concentrations, respectively. These physiological signals are processed in real time to extract biomarkers reflective of cortical excitability and functional state. Real-time signal-analysis algorithms analyze these biomarkers to dynamically adjust TMS parameters (e.g., stimulation intensity, frequency, or orientation) in a personalized manner. This adaptive approach enhances treatment efficacy by ensuring that stimulation is delivered during optimal brain states, thereby maximizing neuromodulatory effects and improving clinical outcomes. TEPs, TMS-evoked potentials.

### 4.2 Device Development

Device innovation is crucial for translating advanced rTMS protocols into clinical practice. Current rTMS systems face two major technical limitations: insufficient targeting accuracy and stability (due to head movement during sessions), and large, cumbersome equipment (typically confined to hospital or specialized clinic settings). To address the former, robot-assisted TMS (e.g., Robo-TMS) systems have been developed that improve targeting precision and stability through automated positioning and real-time tracking [137,138]. Although current clinical application is limited by operational complexity, practicality, and cost, technological advances are expected to mitigate these issues [137]. The improvement of accessibility, device miniaturization, and portability are key development trends. For instance, a recent study reported a battery-powered, wearable suprathreshold rTMS device that maintained comparable stimulation intensity and frequency to conventional commercial systems, and reduced total weight to approximately 3 kg and power consumption to only 10% of traditional devices [139]. This design allowed treatment during free movement, potentially enabling convenient home- and community-based rTMS therapy [139]. The continued development and optimization of such portable devices will significantly enhance the accessibility and convenience of rTMS treatment, facilitating its broader application.

## 5. Discussion

rTMS has established its position as a safe and effective non-invasive neuromodulation therapy for depression, particularly for TRD. Moving beyond standard protocols, the field of rTMS is currently undergoing a profound transformation centered on precision and efficiency. Through functional target navigation, individualized parameter setting (e.g., intensity, pattern), innovative stimulation protocols (such as TBS, pTMS, and accelerated regimens), and the exploration of closed-loop systems, researchers are striving to enhance the efficacy, efficiency, and response rates of rTMS. The application of individualized functional targeting and accelerated TBS protocols has demonstrated clear clinical value [83,84,91,98].

However, translating optimized laboratory findings into widespread and affordable clinical practice remains a core challenge. The high costs associated with precision targeting [73,83], insufficient standardization of novel treatment protocols [99,100], bottlenecks in closed-loop technology [123,128], and issues regarding device accessibility [137,139], urgently need to be addressed. Future research should focus on: (1) reducing costs and improving feasibility by developing cost-effective alternative solutions for targeting and monitoring; (2) strengthening validation and standardization by confirming best practices for novel protocols (especially accelerated regimens) through large-scale randomized controlled trials; (3) overcoming technical barriers by advancing research on biomarkers and algorithms necessary for closed-loop systems; (4) promoting device innovation by developing miniaturized, portable, and automated devices. It is important to note that there is a need for more effective research to evaluate the outcomes and cost-effectiveness of optimized strategies in real-world settings, moving beyond mere efficacy research.

Since patients in actual clinical practice often receive multimodal treatments, research on integrating rTMS optimized protocols with psychological and other non-pharmacological methods represents another research direction [140]. For example, multiple randomized controlled trials have demonstrated that the therapeutic efficacy of combined rTMS and tDCS treatment surpassed that of either modality alone and also exceeded sham stimulation [141,142]. However, a recent scoping review and meta-analysis focusing on rTMS combined with psychological and other non-pharmacological methods (such as aerobic exercise, bright light therapy, and cognitive training) concluded that such combinations have not yet consistently outperformed rTMS alone [143]. This discrepancy may have stemmed from differences in the type of adjunctive intervention, study design, or patient sample. Specifically, differential neuromodulation combinations (e.g., rTMS + tDCS) may have engaged complementary physiological mechanisms, whereas rTMS combined with behavioral or psychological interventions may have involved more variable and context-dependent synergies.

Such discrepancies, in which individual trials show promise but meta-analyses remain inconclusive, are not unique to multimodal treatments but are prevalent across novel rTMS interventions. These conflicting findings likely stemmed from substantial heterogeneity in study designs, patient neurophysiologies, and the lack of standardized biomarkers. For instance, a protocol that fails in a broad, unstratified sample may prove highly effective in a specific biotype of depression characterized by distinct connectivity patterns. Therefore, reconciling these conflicting results would require a paradigm shift from ‘one-size-fits-all’ efficacy trials to biomarker-stratified studies. Future clinical guidance should prioritize the matching of specific stimulation parameters to individual neurophysiological profiles, thereby reducing variability and improving clinical translation.

## 6. Conclusions

Although standard rTMS is an effective treatment for depression, its clinical utility is being significantly expanded by recent optimization strategies. With the advancement of precision medicine and rapid progress in neuroscience technologies (e.g., neuroimaging, computational modeling, biosensing, and artificial intelligence), rTMS is poised to overcome current limitations regarding efficiency and variability. Future rTMS therapy is expected to become smarter (closed-loop adaptive), more efficient (reliable accelerated protocols), more precise (based on individual brain network states), and more accessible (portable devices). Ultimately, optimized rTMS will evolve from an important adjunctive treatment into a powerful pillar within the depression treatment framework, offering faster, more effective, and more individualized recovery hope for a greater number of patients.
